# *Bakterion*: development of a serious game for microbiology education

**DOI:** 10.1590/1414-431X2025e14866

**Published:** 2026-01-30

**Authors:** L.R. Campos, H.V. de Morais, G. Seabra, K.V. dos Santos, D.C.S.A. Araújo

**Affiliations:** 1 Programa de Pós-Graduação em Assistência Farmacêutica, Universidade Federal do Espírito Santo, Vitória, ES, Brasil; 2 Laboratório de Inovação para o Cuidado em Saúde, Universidade Federal do Espírito Santo, Vitória, ES, Brasil; 3 Programa de Pós-Graduação em Biotecnologia, Universidade Federal do Espírito Santo, Vitória, ES, Brasil; 4 Núcleo de Bioengenharia Tecidual, Universidade Federal do Espírito Santo, Vitória, ES, Brasil; 5 Programa de Pós-Graduação em Doenças Infecciosas, Universidade Federal do Espírito Santo, Vitória, ES, Brasil; 6 Laboratório de Resistência Bacteriana, Universidade Federal do Espírito Santo, Vitória, ES, Brasil; 7 Programa de Pós-Graduação em Ciências Farmacêuticas, Universidade Federal do Espírito Santo, Vitória, ES, Brasil; 8 Laboratório de Biologia de Microrganismos e Antimicrobianos, Universidade Federal do Espírito Santo, Vitória, ES, Brasil

**Keywords:** Serious games, “*Game Design Thinking*”, Microbiology, Bacterial resistance, Antimicrobials

## Abstract

Microbiology education is traditionally lecture-based, with few studies exploring active methodologies such as serious games. In this context, this study aimed to develop a serious card game, ‘*Bakterion'*, as a teaching tool for antimicrobial education. The game was designed based on *Game Design Thinking*, following the stages of Empathy, Ideation, and Implementation. Inspired by the game *Munchkin^®^*, *Bakterion* includes a rulebook, markers, and 200 cards featuring illustrations based on electron microscopy and laboratory materials. The cards were designed to be easy to use, allowing players to correlate elements even without prior specialized knowledge. The game presents an innovative approach to microbiology education, fostering student engagement and active participation. *Bakterion* may serve as a promising tool for teaching microbiology and antimicrobial resistance, complementing traditional methods. Future studies should assess the impact of *Bakterion* on student learning.

## Introduction

The teaching of microbiology in undergraduate education plays a crucial role in the training of healthcare professionals (1). However, assimilating the associated concepts can be challenging for students due to the fragmentation of disciplines, the complexity of microbial structures and mechanisms (2), and the lack of connection between microbiology and everyday life (3). Additionally, the absence of adequate instruction on microorganisms (2) and the limited encouragement of innovative methodologies may hinder learning (2). Traditionally, microbiology education has been through lecture-based classes, where the professor acts as the primary transmitter of knowledge, while students assume a passive role, limiting the learning process to the mere reproduction of content (4,5).

Active learning methodologies aim to transform the educational process, shifting students from a passive role, primarily as listeners, to an active role as protagonists in constructing their own knowledge (5,6). Studies indicate that these methodologies promote greater engagement, improved learning, and enhanced academic performance across various fields, including science, engineering, mathematics, and microbiology (7). Among active learning methodologies, serious games have been widely employed in the health field, encompassing various specialties, including microbiology teaching (8).

Serious games stand out as an approach in which the primary goal is not entertainment, but rather knowledge acquisition, training, or behavioral change (9,10). Although fun is not the central focus, these games can nonetheless engage or entertain players. Beyond their playful aspect, other fundamental characteristics such as well-defined objectives, clear rules, stimulating challenges, interaction, continuous feedback, and an engaging narrative contribute to the effectiveness of serious games (11,12). For these games to fulfill their educational purpose, it is essential to consider factors that foster both learning and engagement, including identifying the target audience and content, game attractiveness, ease of use, effective interactions (user-user, user-game, and user-instructor), clarity of objectives, stimulation of critical thinking, adoption of new perspectives, and reward mechanisms (13).

Serious games have gained increasing visibility in recent years, with applications in scientific education and graduate programs focused on drug development (14). Recognized as a promising pedagogical tool, they can be applied in various learning environments, both in formal and informal contexts (12,13,15). In healthcare education, serious games have shown positive results in areas such as obstetrics, pharmacology, and intensive care, enhancing learning and professional training (12). In the context of microbiology, the objective of these games goes beyond merely transmitting knowledge about the relationship between diseases and microorganisms; they seek to foster a reflective approach to the teaching-learning process, making education more meaningful and stimulating logical reasoning (2).

Despite the increasing use of serious games in higher education, particularly in the health sciences, gaps remain in the field of microbiology, where only a few studies have explored their application (2). A literature review conducted by Nowbuth et al. (16) found six studies that used digital games to enhance the training of future health professionals. Edwards et al. (8), in a more recent and comprehensive scoping review, identified a wide range of analog games developed for medical education, including those specifically focused on microbiology and infectious diseases, such as *The Antimicrobial Stewardship Game*, *Bacteria Game*, and *Doc in the Box*. Although these games address central topics such as antimicrobial resistance, rational use of antibiotics, and clinically relevant pathogens, their mechanisms are quiz-based or case-based, requiring prior knowledge from players.

In contrast, a key distinguishing feature of the serious game developed in this study - *Bakterion* - is that it requires no prior knowledge to play and is designed as a strategic and integrative analog game. Its primary goal is to support microbiology learning of health science students dynamically and engagingly, while also fostering critical clinical skills such as the rational use of antimicrobials. To this end, the game incorporates essential microbiological concepts directly into its components - such as identification of bacterial morphology, infection types, antimicrobial susceptibility, and classification - allowing players to learn through active decision-making and content exploration.

Therefore, this article aimed to describe the development process of *Bakterion*, a strategic analog card game designed to support the teaching of microbiology and antimicrobial stewardship for health science students. The study details the stages of design, prototyping, and initial testing of the game, highlighting its educational objectives, mechanics, and potential applications within active learning methodologies.

## Material and Methods

The *Bakterion* game was developed using the *Game Design Thinking* methodology, which combines the concepts of game design and design thinking (17). Game design establishes processes, key factors, and game characteristics to be addressed and developed by the team (18), while design thinking consists of a set of structured design strategies that establish a creation process - not merely cognitive but also a production method (19). In recent years, design thinking strategies have been adapted by many authors to guide both learning and game development (17,18,20).

The development of *Bakterion* followed a design thinking approach structured into three phases: I) Inspiration, which involved identifying the need for an educational tool to support microbiology teaching, especially regarding antimicrobial resistance; II) Ideation, in which ideas for the game's theme, mechanics, and educational content were generated and refined; and III) Implementation, where prototypes were created, tested, and iteratively adjusted based on qualitative feedback from the development team and experienced board game players. The process was guided by design thinking principles focused on user needs, idea generation, and iterative improvement to support learning outcomes (17-20).

### Empathy and inspiration

Bridges of insight are built through empathy, attempting to see the world through others' experiences and feel through their emotions (19). The mission of *design thinking* is to translate observations into insights, and insights into new ways to improve people's lives (19). In the context of empathy, the goal is to establish individuals' perspectives on problems, as well as evaluate possible solutions in ways that maximizes their benefits (19).

In this regard, to broaden the perspective on potential topics to be addressed, an analysis of the Pedagogical Course Projects (PPCs) from the Health Sciences Center (CCS) at the Federal University of Espírito Santo, Maruípe campus, was conducted. This analysis included the courses in nursing, pharmacy, physiotherapy, speech therapy, medicine, nutrition, dentistry, and occupational therapy. The focus was to identify common subjects among these programs that cover essential topics for student training. It was observed that only the occupational therapy program does not have microbiology-related courses as mandatory components.

The microbiology discipline, present in most health-related programs, was chosen as the overarching theme of the project. This decision was based not only on its curricular scope but also on its practical relevance, considering global health challenges such as the rise of antimicrobial resistance (AMR) and the inappropriate use of these medications (21). The experience of the COVID-19 pandemic further highlighted the importance of microbiological knowledge, particularly regarding issues related to medication misuse (21).

Within this broader discipline, the field of bacteriology was prioritized as the specific thematic axis of the game. This choice was justified by its central role in raising awareness about the rational use of antimicrobials and promoting preventive health practices, given the global AMR problem (22) and the risks associated with the irrational use of these medications, which can increase selective pressure and favor the emergence of resistant strains (22,23). These concerns were especially pronounced during the COVID-19 pandemic years (24).

During this phase, the goal was to create an educational tool that complemented traditional teaching, offering a format aimed at increasing the active and meaningful engagement of students, without losing focus on methodological rigor. The choice of a serious game as a teaching and learning strategy was intended to balance the need to optimize pedagogical resources with the importance of promoting accessible, efficient, and more attractive learning, aligned with students' cognitive demands. Thus, the game does not serve as a substitute for conventional teaching but as an extension that helps reinforce and consolidate innovative and practical learning (25).

### Definition and ideation

After the empathy and inspiration phase, the results obtained were used to define the topics to be addressed in the game. Several topics on bacteriology were proposed by the game developers, including: bacteria, the use of antimicrobials, and their mechanisms of action, identification and isolation, signs and symptoms of infections, metabolism and genetics of pathogenic bacteria, among others.

Based on the selected themes, the information was organized in a Microsoft Office Excel^®^ spreadsheet, which was used to create the integrated parts of the project, with the following reference books: Antimicrobials and Chemotherapeutics for the Clinician (26), Microbiology 6th Edition (27), Medical Microbiology 7th Edition (28), and the documents “Intrinsic Resistance and Rare Phenotypes Version 3.2” (29) and “CLSI M100-ED32:2022 Performance Standards for Antimicrobial Susceptibility Testing, 32nd Edition” (30). With this information, the development of the game methodology began.

During the development and ideation process, modifications were made to improve both the inclusion of relevant content and the gameplay. This process was divided into two main stages.

The first stage, called divergent ideation, involved generating various proposals and ideas for different approaches, mechanics, and forms of interaction that could be suitable for the content selected for the game. At this point, two main proposals emerged: i) a game centered around a shared main board, with individual boards for each player to develop their strategy. The goal would be to relate antimicrobials to their mechanisms of action and identify susceptible bacteria; ii) a card-based game, where each card provided information about bacteria, culture media, and antimicrobials, allowing for battles between players through the combination of strategic cards.

The second stage, convergent ideation, involved evaluating the alternatives generated in the previous stage, with the aim of identifying and refining the best available options (19). This process was essential because the analysis and objective testing of competing ideas increase the likelihood of achieving innovative results, often stemming from the combination or improvement of different proposals (20). In this stage, both ideas were initially developed in greater detail. However, the proposal based on boards was discarded. The limitations in the game mechanics and the scope of the content that could be explored in this approach indicated that the second idea, centered on the deck of cards, was more versatile and sui for achieving the proposed educational objectives.

The dynamics of the game Munchkin^®^ was used as inspiration; consequently, the game's goal would be to eliminate bacteria - ludically illustrated as monsters - through the use of antimicrobials. It is important to note, however, that this representation is intentionally focused on harmful, pathogenic bacteria, which are the target of antimicrobial therapy in clinical practice. This distinction can be further explored by educators when using the game, reinforcing the broader concept that bacteria can establish beneficial, neutral, or harmful relationships with humans. Other elements were added throughout the process, such as hygiene techniques and the use of personal protective equipment (PPE). This stage, prior to prototyping, was crucial for development, as it helps minimize losses by identifying the options that are more likely to succeed in application.

### Implementation: prototyping and testing

The game prototype was developed based on the ideas and results obtained in the previous stages. In this phase, two important activities were carried out: presentation of the prototype and testing of the game with the development team to gather feedback and analyze potential improvements (17). After the team's consensus, three classes of cards began to be designed: Bacteria, Antimicrobials, and Special Cards - PPE and Action.

The “Bacteria Cards” represent the bacteria to be faced and eliminated throughout the game. The “Antimicrobial Cards” represent the antimicrobials used in clinical practice to treat an infected patient. In the game, these cards are used to eliminate bacteria and score a point. The “Special Cards” are those that can be equipped by the player to enhance combat strength and help eliminate bacteria.

For the creation of the cards, graphic resources such as illustrations and iconography were used throughout the project. The drawings were inspired by photographs, illustrations, electron microscope images, and objects routinely used in a microbiology laboratory. These resources were employed through the use of a painting software (PaintTool SAI^®^, Systemax Software Development, Japan), two graphics tablets, image manipulation software (GIMP^®^ - GNU Image Manipulation Program, USA), as well as an online tool to assist with layout and design creation (Canva^®^, Canva Pty Ltd., Australia). The technique of tracing was utilized, a method that involves drawing over other images, separated by layers, in order to give all the images a uniform style. The visual design of the game was conceived and crafted to evoke the school environment, with simpler lines for the illustrations and the use of the Schoolbell font in Canva^®^.

Based on the dynamics of the card game and the division of card types, we analyzed which elements and details, from a microbiological perspective, would make more sense for the learning of laboratory and clinical practices and could be included in each card.

#### Bacteria Cards

The information contained in the Bacteria Cards was presented clearly and objectively to enhance game flow and playability. The information included was: scientific name; site of infection; Gram stain; first-line antimicrobials; AMR; and the points required for combat. Initially, only the intrinsic resistance of bacteria to each antimicrobial was considered (29,30).

From this point, the possibility of adding types of Acquired Resistance was considered. To support this addition, the article “Global burden of bacterial antimicrobial resistance in 2019: a systematic analysis” (31), which describes global resistance patterns up until 2019, was used. According to the article, the resistance patterns for species and types of bacteria up to 2019 included: i) *Escherichia coli* - extended-spectrum beta-lactamase (ESBL) resistance. This resistance was observed in several regions, including Southeast Asia and Sub-Saharan Africa; ii) *Klebsiella pneumoniae* - carbapenem-resistant (KPC) and ESBL. Resistance to KPC was particularly high in regions such as Latin America and Eastern Europe; iii) *Acinetobacter baumannii* - carbapenem resistance, with high prevalence in areas such as the Middle East and Africa; iv) *Staphylococcus aureus* - methicillin-resistant *Staphylococcus aureus* (MRSA), with elevated levels in regions like Northern Europe and North America.

According to the article's analysis, the following bacteria were included in the game: *Klebsiella pneumoniae* (carbapenem-resistant), *Acinetobacter baumannii* (carbapenem-resistant), and *Pseudomonas aeruginosa*. Additionally, ESBL resistance was added to *Escherichia coli*. To complement the deck with other resistant bacteria, *Enterobacter spp.* (AmpC beta-lactamase producers) and *Enterococcus spp.* vancomycin-resistant enterococci (VRE) were also included. This expanded the diversity and realism of the game by addressing various clinically relevant resistance mechanisms.

Gameplay testing was then carried out by the research and development team, defining the game's mechanics and scoring. For each Bacteria Card, a combat value was assigned. Initially, this value was set considering the difficulty of combat in relation to the number of first-line antimicrobials, resistances, and other common antimicrobial classes.

For the testing of the game's mechanics, hypothetical clinical situations were considered, such as patient health conditions that limit the use of certain antimicrobials, such as pregnancy or nephropathy. Also included in the bench deck were the “Antibiogram” and “MacConkey Agar” Cards. The first card illustrates the effect of antimicrobial sensitivity testing using the antibiogram technique. The second card illustrates the selectivity of MacConkey Agar, a culture medium used to isolate and differentiate Gram-negative bacteria.

The bacteria's “life” values (Bacterial Strength) were defined based on their clinical severity, AMR, and the effectiveness of available treatments in a way that gameplay and adherence to the real-world public health context were balanced. Bacteria such as KPC and *Pseudomonas aeruginosa* were assigned high values due to their high resistance and the need for last-line antimicrobials for treatment (32), while *Clostridium difficile* and *Helicobacter pylori*, with effective treatments available, were given lower values (33).

To define these parameters, artificial intelligence (AI) (ChatGPT, OpenAI) was used to cross-reference the information from scientific literature and clinical data regarding antimicrobial resistance profiles, prevalence, clinical severity, therapeutic efficacy, and epidemiological relevance for each bacterium (34).

The process can be explained as follows:

Phase 1: Data collection: AI was fed with scientific information on bacterial resistance, prevalence, and treatment efficacy from articles, guidelines, and epidemiological reports (26-31).

Phase 2: Classification: The data were categorized by clinical severity, resistance mechanisms, recommended antimicrobials, and therapeutic efficacy, identifying relevant patterns.

Phase 3: Parameter definition: AI applied criteria such as prevalence, AMR, clinical severity, therapeutic efficacy, and epidemiological relevance for each bacterium. The prompt used in this phase was as follows: “Consider that I am creating an educational game about bacterial infections. I need to assign a numerical value called ‘life value' to each bacterium. This value represents the difficulty of combating the infection on a scale from 1 to 20 and should be based on the following criteria: a) antimicrobial resistance (e.g., multidrug-resistant, carbapenem-resistant, ESBL-producing, etc.); b) clinical severity of the infection (ability to cause death, prolonged hospitalizations, etc.); c) effectiveness of available antimicrobials (whether there are still effective treatment options or if treatment is extremely limited); d) prevalence and number of reported cases since 2019, especially in relevant healthcare-associated or community infections; and e) tissue or body system affected (e.g., respiratory, urinary, central nervous system, etc.). Based on these variables, generate a table with the estimated life value for each bacterium in the list below and briefly justify each assigned value. List of bacteria: ‘Carbapenem-resistant *Acinetobacter baumannii*; ESBL-producing *Escherichia coli*; KPC-producing Klebsiella pneumoniae; MRSA (methicillin-resistant *Staphylococcus aureus*); *Pseudomonas aeruginosa*; etc.”

Phase 4: Score calculation: based on the parameters, AI determined the “life” value of the bacteria, balancing scientific accuracy and gameplay.

Phase 5: Cross-validation: the proposed values were compared with real-life clinical treatments (35) and feedback obtained from initial game tests, refining the AI's assignments to ensure they were aligned with practical context.

#### Antimicrobial Cards

The Antimicrobial Cards were designed using a color-class system. Different colors were selected for each class to optimize gameplay and stimulate student learning through color association. The selection of antimicrobials for the game prototype was carried out based on specific criteria to ensure the relevance and applicability of the information. Initially, 80 antimicrobials distributed across 22 different classes, such as aminoglycosides, cephalosporins, fluoroquinolones, penicillins, among others, were selected.

To make the game more focused and practical, the selected antimicrobials underwent a thorough review. The first filter excluded drugs that were unavailable or not commonly used in Brazil (26,31). In the second stage, antimicrobials targeting bacteria not represented in the deck were removed, ensuring alignment between treatments and the pathogens included in the game. Finally, the selection prioritized first-line antimicrobials recommended for the treatment of bacterial infections in Brazil. When combined, these agents offer coherent and effective therapeutic strategies within the proposed clinical scenarios, reflecting treatments consistent with national clinical practice.

This process ensured that the final deck contained only relevant antimicrobials, enabling coherent and effective treatment strategies within the proposed clinical scenarios. After revisions, the new deck was reduced to 27 types of antimicrobials, selected to offer a more efficient dynamic and align with the game's context. This allowed the inclusion of additional information, such as the activity spectrum of each antimicrobial and strategic combinations that generated bonuses for the player during combat.

The design of the cards was adjusted to accommodate this new information, preserving details about the mechanism of action of each antimicrobial. Additionally, the dynamic of antimicrobial strength was restructured. In the initial tests, each antimicrobial had a fixed value of 1 point in combat, while the antimicrobials of choice indicated on the Bacteria Card received an extra point. After the revision, the strength became specific to each antimicrobial, considering factors such as resistance profiles, sensitivity, and the clinical context, providing the game with a more realistic perspective.

#### Special Cards - PPE and Action

The Special Cards included the PPE Cards, which feature an image of the item, its name, and the corresponding action points, as well as the Action Cards. The Action Cards were designed as interference cards. Each card has its own action, such as discarding a PPE, transferring a bacterium for another player to confront, or stealing an item from an opponent. The Action Cards are described in Table 1.

**Table 1:** Description of the “Action Cards” developed for the *Bakterion* game.

Types of cards	Description of the cards
“Nephropathy”, “Pregnant”, “Hepatopathy” and “Newborn” Patients	These cards simulate clinical profiles in which bacterial infections must be treated, indicating specific restrictions on the use of certain antimicrobials.
Discard	Discard an item the player is equipped with.
Bunsen Burner	Grants a temporary bonus in combat.
Inoculating Loop	Used to transfer the microorganism to another player to battle or collect a microorganism in play for your own battle.
Lab tweezers	Used to steal an item from another player.
Autoclave	Used to discard a bacterium in combat and flee from the fight.
Mixed Flora	Used to add an extra bacterium to the battle.
Log Phase	The microorganism gains 2 strength points in combat.
Decline Phase	The microorganism loses 2 strength points in combat.
Technician	The player gains a 1-point bonus in combat.
Microbiologist Specialist	The player gains a 2-point bonus in combat.
Virulence Factors	Increases the strength of the bacteria if it produces the described factor, aiming to make the game more competitive and educational.
Versatile Antimicrobial	Functions as a wildcard that can be used to duplicate the effect of an antimicrobial or as part of a synergistic combination, providing a combat bonus.
Immune Support	Represents interventions that strengthen the patient's immunity (such as serums, vitamins, or vaccines), reducing their vulnerability to infection.

For the application of the tests, 68×94 mm cards were printed on 230-g color photographic paper. The description of the decks is shown in Table 2.

**Table 2:** Description of the Bench and Stock Cards developed for the *Bakterion* game.

	Number of cards
Bench Deck	
Discard Cards	8
Patient Cards (Pregnant Women/Newborn/Nephropathy/Hepatopathy)	8
PPE/Good Laboratory Practice Cards	12
Bacteria Cards	18
Antibiogram Cards	2
MacConkey Agar Cards	2
Technician Card	1
Experts Card	1
Versatile Antimicrobial Card	1
Stock Deck	
Antimicrobial Cards	72
Experts Cards	3
Technician Cards	3
PPE/Good Laboratory Practice Cards	36
Discard Cards	4
Bonus Cards (green)	14
Virulence Factor Cards	6
Mixed Flora Cards	3
Log Phase Cards	3
Decline Phase Cards	3

#### Validation based on player experience

The test round involved four players and two developers, with one developer actively participating as a player and the other serving as mediator and observer. Throughout the session, participants shared observations regarding key aspects such as rule clarity, the functionality of visual and textual elements on the cards, and the overall flow of gameplay. These insights were instrumental in identifying potential adjustments to enhance both the educational value and the gaming experience.

Feedback was collected from experienced board game players during this test round, guided by the developers. Participants were asked to evaluate specific criteria, including clarity and objectivity (comprehension of rules, texts, and game mechanics), design (esthetics, organization, and usability of cards and graphic elements), card content (relevance, coherence, and accuracy), and gameplay dynamics (move fluidity, player engagement, and balance between challenge and enjoyment).

Qualitative observations and concrete suggestions concerning card mechanics and text content were gathered during gameplay. After the session, participants completed a structured form to provide their perceptions on these aspects. The responses were analyzed to identify recurring themes, suggest improvements, and validate the game's design and dynamics. A summary of the players' improvement suggestions is presented in Table 3.

**Table 3:** Suggestions for improvements obtained after the pilot testing round.

Category	Suggestion	Detailed description
Specific Cards	“Versatile antimicrobial” card	Review text and mechanics to make the statement “acts as an antimicrobial of choice or combination” clearer.
Start of Game	Initial briefing	Players start as students (1 experience point) and progress to technician (2 points) and Experts (3 points).
Fighting Bacteria	Requirements for victory	The player must overcome the strength of the bacteria. Experts win draws, increasing the value of the title.
Disposable Cards	Loss of antimicrobial cards	All used cards must be discarded after combat, reinforcing the concept of single-use antimicrobials.
PPE and Bonuses	Use of disposable PPE	PPE is discarded after use in combat, but victory grants 1 additional experience point to the player.
Visual Adjustments to Cards	Spectrum of action	Indicate more clearly the predominant Gram type in antimicrobial charts.
	Virulence factor	Visually highlight the “additional strength” value on cards to make it easier to read during play.

PPE: personal protective equipment.

Among the implemented improvement suggestions were: i) Revision of the “Versatile Antimicrobial” Card text: the text was adjusted for greater clarity to: “Can be used as any antimicrobial, alone or in combination.”; ii) Change in victory conditions: previously, the player could win by matching their strength with the bacterial life force. After the change, only players with the “Specialist” title can win in case of a tie in strength; iii) Alteration in antimicrobial loss rule: previously, when losing a battle, the player would recover the antimicrobials used, losing only the victory point. After the change, all cards used in the battle are discarded; iv) Redesign of the “Virulence Factor” Cards: the design was enhanced to highlight the additional bacterial strength value, improving readability and making gameplay more fluid.

Some suggestions were temporarily discarded as they would negatively impact the game dynamics. For example, the idea of including the discard of EPIs would require recalculating and increasing the number of these cards in the deck to ensure players could recover them. However, this would make the game longer and less dynamic. Similarly, the suggestion to highlight the predominant Gram type for each antimicrobial could complicate card reading, diverting the focus from the primary objective: encouraging players to prioritize first-line antimicrobials, rather than focusing solely on the Gram type and spectrum of action, which is secondary information.

## Results

### Composition of the *Bakterion* game

**Figure 1:**
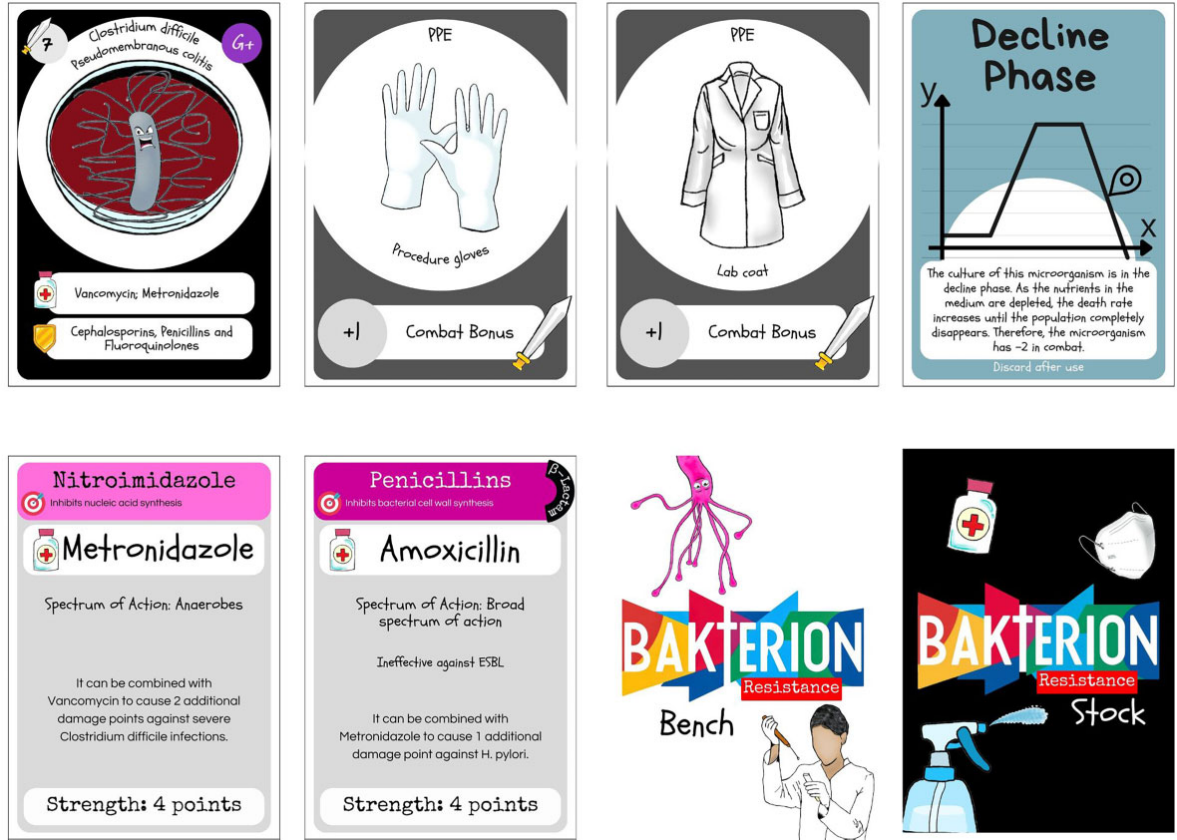
Examples of the cards designed for the *Bakterion* game. The *Clostridium difficile* Bacteria Card displays the related disease, first-line antimicrobials, and types of resistance. The PPE (personal protective equipment) Cards show their respective bonuses. The Decline Phase Action Card can be played to weaken the bacteria in combat. The Antimicrobial Cards characterize the antimicrobial name and class, as well as their Strength and respective spectrum of action. The back designs of the Bench and Stock Cards are also shown.

The game consists of 54 point markers, printed on 230-g color photographic paper, measuring 30×30 mm, a rulebook (Appendix 1), and two decks totaling 200 cards (exemplified in Figure 1) printed on 230-g color photographic paper, measuring 68×94 mm. These cards include: 10 different types of bacterial species: *Acinetobacter baumannii* resistant to carbapenems (CRAB); *Clostridium difficile*; *Enterobacter spp.* AmpC beta-lactamase; *Enterococcus spp.* resistant to vancomycin (VRE); *Escherichia coli* producing extended-spectrum beta-lactamase (ESBL); *Helicobacter pylori*; *Klebsiella pneumoniae* producing carbapenemase (KPC); *Pseudomonas aeruginosa*; *Salmonella Typhi*; *Staphylococcus aureus* resistant to methicillin (MRSA). Each bacterium has its own combat strength, which is indicated in the upper left corner of the card; 1 Antibiogram Card - Grants the player 1 victory point when using an antimicrobial, illustrating the effect of antimicrobial sensitivity in the antibiogram technique; 1 MacConkey Agar Card - Grants the player 1 victory point, illustrating the selectivity of the agar; 1 Specialist Card; 1 Technician Card; 11 different types of PPE/Good Laboratory Practices Cards, totaling 48 cards - These cards grant the player “Combat Strength” points; 27 different types of Antimicrobial Cards; some cards were duplicated, triplicated, or quadrupled for balance and difficulty in the game, totaling 72 Antimicrobial Cards - These cards grant the player “Combat Strength” points; 4 different types of Discard Cards; 4 different types of Bonus Cards; 4 different Patient Cards; 3 different Virulence Factor Cards; 3 Mixed Flora Cards; 3 Log Phase Cards; and 3 Decline Phase Cards.

### *Bakterion* game mechanics

The game *Bakterion* revolves around combating bacteria using antimicrobials following the Good Laboratory Practices. It was designed for three or more players, allowing constant interaction through discussions about actions, assistance, and card exchange. There is no established maximum number of players, although it has been tested with groups of up to eight players. However, for a smoother dynamic and better gameplay experience, a maximum of six players is recommended.

The game consists of two decks of cards: a Stock Deck (containing Antimicrobial Cards, Good Practices/PPE Cards, and Bonus cards) and a Bench Deck (containing Bacteria Cards, Action Cards, and Bonus Cards). These decks must be shuffled separately, always maintaining two discard piles - one for Bench Deck cards and one for Stock Deck cards. When a draw deck is depleted, the corresponding discard pile can be reshuffled to form a new draw pile.

**Figure 2:**
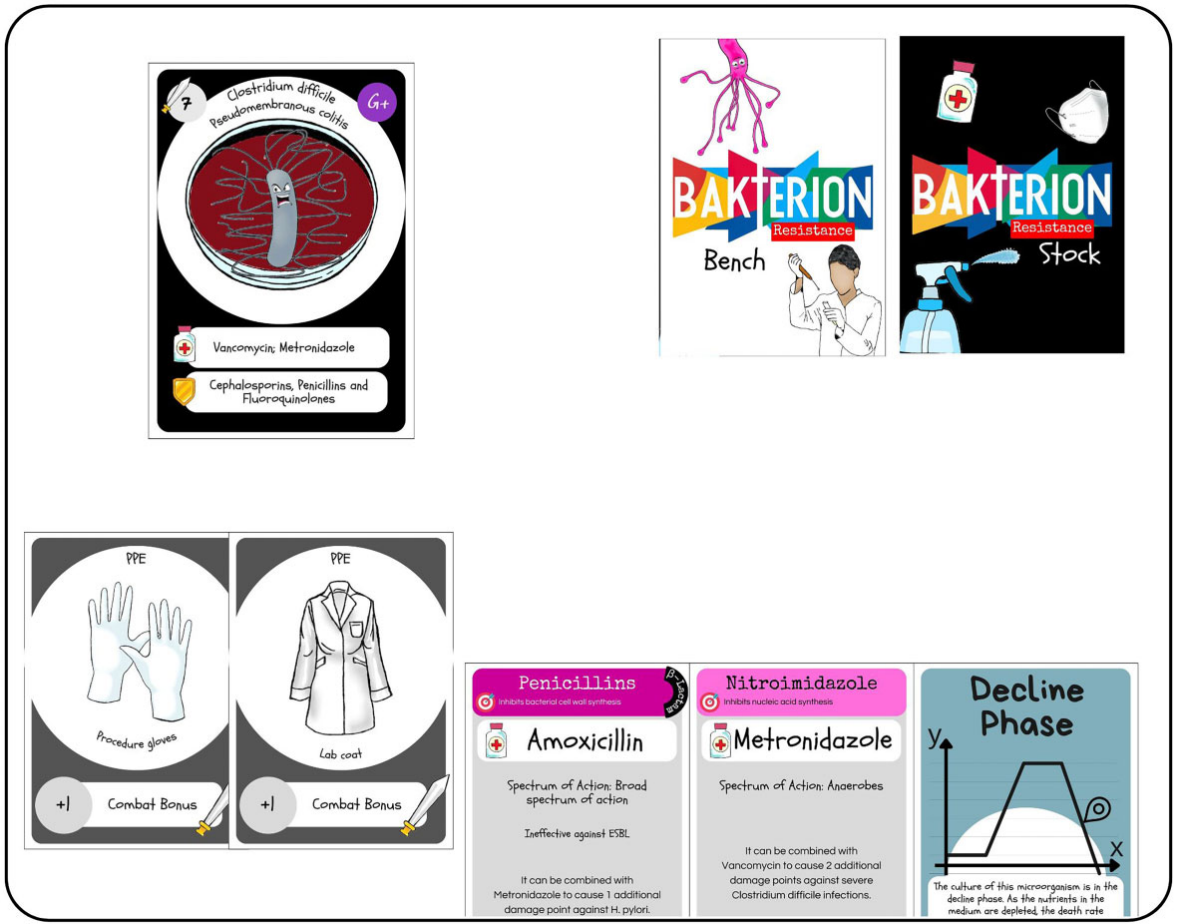
Organizing the cards on the table. The figure illustrates 8 cards from the *Bakterion* game: a Bacteria card, representing a resistant species in a Petri dish; the two decks (Bench and Stock) used in the game; two Personal Protective Equipment (PPE) cards - gloves and lab coat - which provide combat bonuses; two antimicrobial cards (Amoxicillin and Metronidazole), with spectrum of action and strength in points; and a Bacterial Phase Card (“Decline Phase”), which represents the decline phase of the bacteria. After distributing the cards, players may play and equip their Laboratory Best Practices Cards, and arrange their Antimicrobial Cards on the game table as they prefer. Only then does the round begin with the drawing of a card from the Bench Deck.

**Figure 3:**
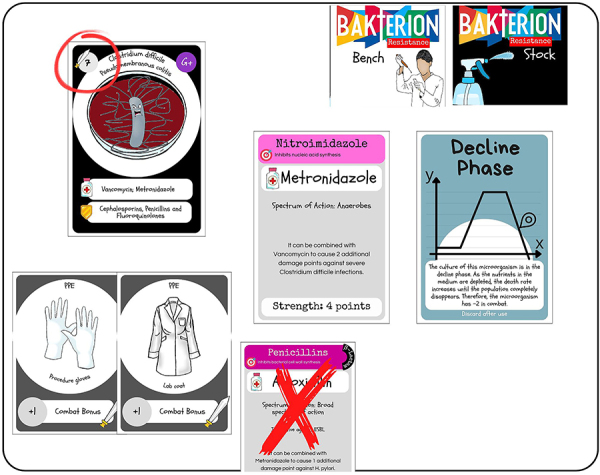
Fighting the bacteria. The figure illustrates a play where the player faces a bacteria using Personal Protective Equipment (PPE) and the antimicrobial metronidazole, while the amoxicillin card (crossed out) is prevented from being used. Activating the “Decline phase” card indicates that the bacteria are in the process of dying.

After the cards are distributed, players may place their Good Laboratory Practices Cards and arrange their Antimicrobial Cards in any preferred order, as in Figure 2. On their turn, each player draws a Bench Deck card from the face-down pile in the center of the table. If the drawn card is a Bacteria Card, the player must engage in combat, as in Figure 3. If the player wins the combat, they recover the number of antimicrobials used plus one from the face-down Stock Deck and earn one point. If the player loses the combat, they lose one point and do not draw any cards.

If the drawn card is not a Bacteria Card, the player must carefully read the card: a) If it is a Patient Card, it remains face-up in front of the player, and its conditions will apply to their next combat; b) If it is a Discard Card, the player must discard whatever is indicated on the card. After drawing the card - whether they discard it or not - their turn ends; c) All other cards can be used by the player at any moment.

If the drawn card is not a Bacteria Card, the player has the option to play one of their Bacteria Cards to initiate combat and earn a point. If the player chooses not to play a Bacteria Card, they may draw two cards, selecting either one from the Bench Deck and one from the Stock Deck or two from the Stock Deck.

Any drawn card is added to the player's hand. If a face-down Bench Deck Card - such as a Bacteria or Discard Card - is drawn into the player's hand, it may later be played against another player. Players can trade cards at any time during the game, with the only restriction being that Antimicrobial Cards can only be traded if they have different mechanisms of action.

To win a combat, the player may combine bonus values with Antimicrobial values. However, at least one Antibiotic Card must always be used in the battle. The player's total Strength must exceed the Bacteria's Strength to win.

The rest of the game's dynamics depend both on player interaction with the game itself and on interactions between players. The first player to reach five Victory Points wins the game. Additional card details are provided in the rulebook.

## Discussion

*Bakterion* was developed as a strategic serious game designed to facilitate microbiology learning through gameplay, without requiring prior knowledge of the subject. Its core educational objective is to integrate essential microbiological concepts - such as bacterial morphology, antimicrobial classes, mechanisms of action, infection types, and biosafety practices - directly into the game's mechanics. Through dynamic decision-making, associations, and peer discussions, the game aims to promote active learning while raising awareness about rational antimicrobial use and preventive measures in health care contexts.

Unlike many existing analog educational games, *Bakterion* allows players to make immediate associations between antimicrobials, their mechanisms of action, and the bacteria they target through a system of visual cues, colors, images, and concise text on the cards. Additionally, the game introduces players to practical infection prevention concepts, including the correct use of PPE, surface disinfection with 70% alcohol or hypochlorite, proper hand hygiene, and laboratory biosafety procedures. These elements were deliberately incorporated into the gameplay, making them accessible actions that can be frequently applied by all players during the game.

The Bacteria Cards were carefully designed to provide detailed information on antimicrobial susceptibility (15,30), resistance patterns (26,29), and key morphological characteristics, including bacterial shape, spatial arrangement, Gram staining profile, and colony color on differential or non-differential media. This information aims to help students develop skills in bacterial identification, beginning with Gram staining and morphological analysis - essential laboratory practices for differentiating bacterial species and selecting appropriate antimicrobials (22). The game also incorporates clinical microbiology practices, such as the antimicrobial susceptibility test or antibiogram, which evaluates bacterial sensitivity to antimicrobials to allow the selection of effective therapies, preventing treatment failures due to resistant pathogens (22).

The learning objectives of *Bakterion* were not planned to be strictly tied to the curricula of specific undergraduate courses. Instead, content selection was guided by the game's mechanics and supported by key references in microbiology education. In some cases, practical adjustments were necessary - for example, the exclusion of combined antimicrobial therapies such as amoxicillin with clavulanate, as this would complicate associations within the game structure. An exception was made for the combination of sulfamethoxazole and trimethoprim, which are commonly tested and used in combination (22,23).

Considering the limited availability of analog serious games for microbiology education, as highlighted by Nowbuth et al. (16) and Edwards et al. (8), *Bakterion* was designed to address specific pedagogical gaps. While existing games, such as the Antimicrobial Stewardship (AMS) Game (36), effectively promote discussion on antimicrobial prescribing, they typically rely on players' preexisting knowledge. In contrast, *Bakterion* was specifically designed to promote learning during gameplay, even among players with limited or no previous exposure to microbiology. Its game mechanics allow students to progressively acquire knowledge through exploration, association, and interaction with the game components. This approach aligns with contemporary principles of active learning, encouraging players to critically analyze information and collaboratively construct knowledge through play.

Finally, although the preliminary testing phases with the development team and experienced board game players provided valuable qualitative feedback for refining the game's mechanics and structure, the limited number of participants represents an important limitation. Further classroom-based testing, involving larger and more diverse groups - particularly undergraduate students unfamiliar with board games - will be essential to assess the educational impact, usability, and engagement potential of *Bakterion*. Future studies will also provide opportunities for iterative improvements and for expanding the game with additional Bacterial and Specialist Cards, broadening its scope and applicability in different learning environments.

### Final considerations

*Bakterion* was designed and planned for students in health-related courses that include microbiology in their curriculum. However, its use could also be evaluated in other contexts, such as for high school students, with the aim of sparking interest and drawing attention to the current importance of bacteria in public health, AMR, and the prevention of diseases through prophylactic measures. It is important to highlight that the most engaging and effective learning does not result solely and directly from the use of games in teaching processes. Future testing and evaluations are needed to not only identify the strengths, but mainly the weaknesses of the game in order to correct them, bringing balance to the game mechanics while promoting learning in an engaging and fun dynamic.

## Data Availability

All data generated or analyzed during this study are included in this published article.
